# Book Reviews

**Published:** 1901-12

**Authors:** 


INTERNATIONAL CLINICS. A Quarterly of Clinical Lectures and Especially Prepared Articles on Medicine, Neurology, Surgery, Therapeutics, Obstetrics, Pediatrics, Pathology, Dermatology, Diseases of the Eye, Ear, Nose and Throat, etc. Edited by HENRY W. CATTELL, A. M, M D., of Philadelphia, with the collaboration of John B. Murphy, M. D., of Chicago; Alexander D. Blackader, M. D., of Montreal; H. C. Wood, M. D., of Philadelphia; T. M. Rotch, M D., of Boston; E. Landolt, M D., of Paris; Thomas G. Morton, M. D., and Charles H. Reed, M.D., of Philadelphia; J. W. Ballantyne, M.D., of Edinburgh, and John Harold, M. D., of London. Volume II. Eleventh series. Philadelphia: J. B. Lippincott Company. 1901. [Price, cloth, $2.00 each; half-leather, $2.25 each.]

The contents of this volume relate to therapeutics, medicine, neurology, surgery, obstetrics and gynecology, pediatrics, pathology, diseases of the eye and laryngology, besides an article relating to the pronunciation and definition of some of the newer medical words, the latter written by W. A. Newman Dorland. The contributors of prominence are J. W. Ballantyne, A. Broca, Alfred Fournier, Santiago Ramon y Cojal, John Griffith, Robert Jardine, and Dr. Wlaeff, from abroad; and A. Blackader, John B. Deaver, M. Allen Starr, Henry Sewall, Ludvig Hektoen, and James Tyson, from this country and Canada. The article by Jay F. Schamberg, of Philadelphia, on smallpox is to be particularly commended, and it is fully illustrated by photographs, showing the eruptions in different stages. This volume as a whole is deserving of approbation.

---

A HANDBOOK OF MATERIA MEDICA, PHARMACY AND THERAPEUTICS. Including the physiological action of drugs, the special therapeutics of disease, official and practical pharmacy and minute directions for prescription writing. By SAMUEL O. L. POTTER, A. M., M. D., M. R. C. P., Lond., formerly professor of the principles and practice of medicine in the Cooper Medical College of San Francisco; Author of the "Quiz Compends of anatomy and materia medica," etc.; late Major and Brigade-Surgeon of Volunteers, U. S. Army. Octavo, pp. 950. Eighth edition, revised and enlarged. Philadelphia: P. Blakiston's Son & Co. 1901. [Price, $5.00.]

This work has been too long before the medical profession and too many editions have been published, to admit even of a hint that it is not occupying a place of prominence in the minds of teachers and physicians or in their libraries.

The author says he began this revision at Manila while serving as attending surgeon there at the headquarters of the Eighth Army Corps, and that it contains information gathered at the Philippines. Other new material has been added, while that which became obsolete or unimportant has been omitted, thus retaining the book at or near its original size.

It is made convenient for references by a thumb index to the several divisions of the volume, and cannot fail to receive the continued favor of teachers, undergraduates and practising physicians.

Memoranda of Poisons. By Thomas Hawkes Tanner, M. D., F. L. S. Eighth revised edition, by Henry Leffmann, A. M., M. D., Professor of Chemistry in the Woman's Medical College of Pennsylvania, etc. Philadelphia: P. Blakiston's Son & Co. 1901. [Price, 75 cents.]

It is well for every physician to have at hand a concise list of poisons with absolute treatment for the same, which is precisely what this little volume furnishes. Tanner has long been recognised as authority on the subject, and has revised this edition down to the present time. It should be kept always within easy reach for the emergency which may arise unexpectedly in the practice of any physician.

---

The Diagnosis and Treatment of Diseases of the Nose, Throat, Naso-Pharynx and Trachea. For the use of students and practitioners. By Cornelius G. Coakley, M. D., professor of laryngology in the University and Bellevue Hospital Medical College, New York. New (2d) edition. In one handsome 12mo volume of 556 pages with 103 engravings and 4 colored plates. Philadelphia and New York: Lea Brothers & Co. 1901. [Cloth, $2.75 net.]

Coakley sprang into prominence as a careful teacher and reliable author, with the issuance of the first edition of this manual. It stamped him with both identity marks at once and a second edition has been called for unusually soon,—a surprise almost to the publishers, if not to the author. In noticing in these columns the first edition we predicted success to the book, but we were not quite prepared to note it so soon. However, that may be fortunate for both reader and author, for it insures a complete revision in a department of medicine where the changes are or have been rapid, making prompt revision necessary to keep pace with advances. The author has done this work most thoroughly, and added a new chapter on the affections of the upper air tract in the infectious diseases. We feel sure that this edition will be taken up even more promptly than the first one.

---

RETINOSCOPY. In the determination of refraction at one meter distance with the plane mirror. By JAMES THORINGTON, A. M., M. D., Author of "Refraction and How to Refract;" professor of diseases of the eye in the Philadelphia Polyclinic and College for Graduates in Medicine. Duodecimo, pp. 89; 51 illustrations, 12 of which are colored. Fourth edition, revised and enlarged. Philadelphia: P. Blakiston's Son & Co. 1901. [Price, $1.00.]

The usefulness of this work is well attested by the demand for repeated editions and its translation into French and German. Thorington long has been recognised as authority on refraction and was among the first to give out in clearly written English, complete instruction as to the application of retinoscopy to refractive work. We think he has chosen the title well,—Retinoscopy. It is better than most of the other terms used to express this method of estimating the refraction, because it tells just what is done—namely, that the retina in its relative position to the dioptric media is being studied. In the preparation of this edition the author has carefully reëxamined the whole subject, made additions where necessary and inserted eight new illustrations, thus bringing it abreast of the period in its observations and teachings.

LECTURES ON NASAL OBSTRUCTION. By A. MARMADUKE SHIELD, M. B. (Camb.), F. R. C. S. (Eng.), Surgeon to St George's Hospital, London, and Surgeon in Charge of the Throat Department, etc. Duodecimo, illustrated. Philadelphia: P. Blakiston's Son & Co. 1901. [Price, $1.50.]

These lectures, if read by the general physicians of the country, would prove of vast benefit to the public at large, for they teach the doctrines of scientific though conservative rhinology. The title of the first lecture is, the causes and diagnosis of the principal varieties of nasal obstructions; of the second, the treatment of nasal obstruction; and of the third, the treatment of nasal polypi. These are practical every-day questions that are presented to physicians, and the author deals with them masterfully, though differing in some respects from the usual methods taught in the clinics and text-books. The conditions treated of are so common and withal so annoying, that this separate monograph is especially timely, and we hope it will receive the careful study of specialist, as well as the generalist, for which latter it seems to have been particularly intended.

THE PHYSICIAN'S VISITING LIST FOR 1902. Fifty-first year of its publication. Philadelphia: P. Blakiston's Son & Co.

Although this is the oldest visiting list in the market, it has kept progress with the years, and contains all that the busy physician needs as a reminder in his every day work. The regular edition is published in five varieties containing record space for from 25 to 100 patients a week, and varying in price from $1.00 to $2.25, according to size. There is also a perpetual edition, which can be commenced at any time and used until full; also a monthly edition in which the patient's name need be written but once a month and the whole account may be kept in one place. Each contains the various tables that are so useful for ready reference, and the book is so compact as to take but a minimum of pocket space.

---

3500 QUESTIONS ON MEDICAL SUBJECTS. Arranged for self-examination With the proper references to standard works in which the correct replies will be found. Third edition, enlarged. With questions of the state examining boards of New York, Pennsylvania and Illinois. Philadelphia: P. Blakiston's Son & Co. 1901. [Price, 10 cents.]

These questions have been selected by a medical teacher and writer of experience, with a particular regard for the wants of medical students. The very fact that the previous editions have been exhausted indicates that the book is wanted. This edition has been revised, considerably enlarged, and questions added from the examining boards of several of the larger states.

---

THE DISEASES OF THE RESPIRATORY ORGANS, ACUTE AND CHRONIC. By WILLIAM F. WAUGH, A. M., M. D., Professor of Practice and Clinical Medicine, Illinois Medical College, etc. Pages 221. Chicago: G. P. Engelhard & Company. 1901. [Price, $1.00, net.]

The influence of such books as this is, to state the case conservatively, of doubtful import. It contains many suggestions of value, but only such as are found in other published works. The trend of its therapeutic teaching is toward the infinitesimal, and it appears to have been written with a view to further the sale of alkaloidal tablets.

---

THE MEDICAL RECORD VISITING LIST OR PHYSICIAN'S DIARY FOR 1902. New revised edition. New York: William Wood & Company, Medical Publishers.

This well-known visiting-list is out in its usual good form and presents features that appeal to the family doctor. The contents are well arranged and could not easily be improved upon, embracing as they do practically everything that a physician need carry in his pocket for reference. The regular styles are arranged for 30 and 60 patients a week at $1.25 and $1.50 each. There are three novelties, bound in genuine seal and calfskin, in two volumes, removable wallets, with and without flap. These vary in price from $2.50 to $4.00, according to quality. Names and addresses will be stamped on books at from 25 to 50 cents extra. Postage or express charges will be paid by the publishers. Orders should be placed with promptitude that they may be filled by the beginning of the new year.

---

THE PHYSICIAN'S POCKET ACCOUNT BOOK. Consisting of a manila-bound book of 208 pages and a leather case. By J J. TAYLOR, M. D. Philadelphia: Published by The Medical Council, Twelfth and Walnut Streets. [Price, $1.00, complete; subsequent books to fill the case, 40 cents each, or three for $1.00.]

This book offers a unique system for keeping physicians' accounts, enabling the doctor to have his business office in his pocket. It is arranged in such a manner as to be admissible in court whenever it becomes necessary to prove the charges according to law. Its many features are ingeniously as well as practically arranged, and this new system of book-keeping makes appeal to such physicians as do not employ an accountant or secretary to attend to the business part of their offices. Especially does it seem to us that the system is adapted to the needs of the country physician, who through its means can always carry with him the information necessary to effect a prompt settlement of accounts when miles away from his office.

---

THE MEDICAL NEWS VISITING LIST FOR 1902 Weekly (dated, for 30 patients); monthly (undated, for 120 patients per month); perpetual (undated, for 30 patients weekly per year); and perpetual (undated, for 60 patients weekly per year) Each style in one wallet-shaped book, with pocket, pencil and rubber. Philadelphia and New York: Lea Brothers & Co., Publishers. [Price, seal grain leather, $1 25; thumb-letter index, 25 cents extra.]

A visiting list is a great time-saver for a busy doctor, and has become well-nigh indispensable to the successful conduct of his practice. The book before us is one of the very best lists published and contains all that a professional pocket-book need embrace to make it of the highest usefulness. The first three styles mentioned above contain 32 pages of data and 160 pages of blanks. The 60 patient, perpetual style, contains 236 pages of blanks. Each style is bound in seal grain leather, wallet-shaped, with pencil. The tables are elaborate and contain almost everything necessary to aid in emergencies.

